# Introduction to special topic—estrogenic control of hypothalamic GnRH neurons

**DOI:** 10.3389/fendo.2012.00137

**Published:** 2012-11-15

**Authors:** Henryk F. Urbanski

**Affiliations:** Division of Neuroscience and Division of Reproductive and Developmental Sciences, Oregon National Primate Research CenterBeaverton, OR, USA

In female mammals, sexual maturation is initiated by a change in the release pattern of pituitary gonadotropins, luteinizing hormone (LH) and follicle-stimulating hormone (FSH), and a consequential increase in the secretion of ovarian sex-steroids, estradiol and progesterone. Orchestrating these endocrine changes is a diffuse population of hypothalamic neurons that produce the neuropeptide, gonadotropin-releasing hormone (GnRH). Although the development and functional integration of GnRH neurons with the rest of the central nervous system is still poorly understood, recent progress has been made on several fronts. For example, there is now evidence that some mammalian species express more than one molecular form of GnRH (GnRH-I and GnRH-II), and that the two corresponding GnRH neuronal sub-populations may play different roles in the regulation of reproductive function and behavior. Moreover, through the use of transgenic animal models, neuronal fiber tracing, gene expression profiling, and electrophysiologcal recordings, new insights have been gained into the mechanisms that regulate GnRH release. This volume brings together 10 articles that reflect current thinking about the role of GnRH in mediating estrogenic feedback to the neuroendocrine reproductive axis. The main focus is on the negative and positive actions that estrogens exert within the hypothalamus of mammals, especially around the time of the preovulatory LH surge. However, the articles also present findings from other vertebrate classes, and so provide an intersting comparative perspective. Taken together, the knowledge presented in this volume represents a foundation upon which to develop more effective therapies for pubertal disorders, infertility, and menopause.

The first article (Sower and Baron, 2011) demonstrates the expression of estrogen receptors within the lamprey hypothalamus, suggesting potential feedback interactions between esatradiol and GnRH neurons. Phylogenetically, lampreys are positioned as basal vertebrates, and like many other non-mammalian vertebrates they express multiple forms of GnRH. Consequently, studies in lampreys provide us with important insights into the molecular evolution of estrogenic-GnRH interactions.

A cluster of three articles then focus on hypothalamic neuropeptides that exert a major influences on the secretion of GnRH, and which are thought to mediate the feedback effects of sex-steroids on the reproductive axis. For example, studies in rodents have clearly shown the importance of kisspeptin to both pulsatile and surge modes of GnRH release (Navarro, 2012), while human studies have observed sexual dimorphism in kisspeptin neurons during aging (Hrabovszky et al., 2011). Again, an interesting evolutionary perspective is provided by comparing the steroid sensitivity of kisspeptin neurons in various non-mammalian vertebrates (Kanda and Oka, 2012).

Although the exact mechanism by which estrogens interact with the reproductive neuroendocrine axis is unclear, our general understanding of this issue is neatly summarized by a series of three reviews (Micevych and Sinchak, 2011; Kenealy and Terasawa, 2012; Radovick et al., 2012). Of particular interest is the finding that sex-steroids exert their feedback not only through classic estrogen receptors located in the nucleus, but also more rapidly through receptors located in the cell membrane.

Various physiological functions show adaptations for life in an environment that undergoes marked changes across the 24-h day. For example, in many mammals the timing of the preovulatory LH surge occurs at a very specific time of day. Two articles discuss the interactions between the circadian timing system and estrogen-sensitive neural circuits that regulate GnRH secretion and the preovulatory surge (Sun et al., 2012; Williams and Kriegsfeld, 2012).

Finally, a novel hypothesis is proposed to explain the conundrum of how estrogens appear to exert both negative and positive feedback actions on the reproductive axis (Urbanski, 2012). Data is presented from non-human primate studies to suggest that negative and positive estrogen feedback are two completely separate mechanisms, which are mediated, respectively, by two distinct GnRH neuronal populations. This hypothesis challenges the common assumption that has dominated the field for the past four decades, namely that individual GnRH neurons are relatively homogenous and can mediate both negative and positive estrogen feedback.
